# Hypochondriacal Insanity as a Precursor of General Paralysis

**Published:** 1861-03

**Authors:** M. Baillarger

**Affiliations:** The Salpetriere, Paris


### HYPOCHONDRIACAL INSANITY AS A PRECURSOR OF GENERAL PARALYSIS.

BY M. BAILLARGER, of the Salpetriere, Paris.

General paralysis is a common and most serious phase of mental disease. It attacks patients of all ages, and its progress towards a fatal termination exhibits stages of the most melancholy and humiliating nature. All medical men accord it as being most insidious in its approach. It may be long in becoming fully developed, presenting at first only the most trivial indications, in many cases so trifling as to pass altogether unobserved; and when the malady does at last attract attention, it may be too late for arresting its advance. It is therefore most important to attend to this disease at the very first; and it is with this object that it seems useful to describe the intimate relation existing between the hypochondriacal form of melancholia and general paralysis.

The relation being understood, it becomes one means of detecting the advent of that disease at the very commencement of its attack. It is of importance to distinguish this symptom, as the melancholy accompanying general paralysis very much resembles melancholia in its simple form. The conceptions or illusions of the hypochondriac, however, although of considerable variety, are yet of such a tendency as often to present something of a special character in their nature. The patients believe that their various organs are changed, destroyed, or completely obstructed: they pretend, for example, to have no mouth, no abdomen, no blood—that their gullet is stopped up, their stomach quite full, their bowels shut up; they imagine that their food passes from its ordinary channels—that it gets into their skin, or even their clothes. Four patients believed their body to have become putrid. Many among such are afflicted with hallucinations of smells. Some keep their eyes closed, and allege they are blind; others cease to speak, and state afterwards that it was impossible for them to open their mouth; they assert that they cannot swallow, nor defecate, nor make water; they affirm that their members are altered—that they are larger or smaller; they say they do not exist, or even go so far as to believe themselves dead; they remain motionless, the eyes shut, and when their limbs are lifted they let them fall, as if completely paralyzed. These different delusions lead to serious consequences: many of the patients refuse, more or less obstinately, to take food, and sometimes it becomes necessary to feed them by means of the stomach pump; and such patients speedily become much emaciated. I have seen, says M. Baillarger, a lunatic die in eight days from obstinately resisting the employment of the stomach pump, under the impression that his stomach was completely full, and his gullet obstructed. One patient pretended he could not make water, and used every effort to retain it; his bladder became enormously distended, and he was at last attacked by a veritable retention, and it was with great difficulty the catheter could be used. In the end a false passage was made, and the patient died, while yet in the first period of the disease.

The tendency to gangrene, which is one of the characteristics of general paralysis in its latter stages, exists in these cases markedly, and before its usual period. Four cases had large eschars over the sacrum, without ever having been confined to bed; one woman, who for a year had exhibited all the symptoms of commencing general paralysis, preserved every appearance of health otherwise, when, all of a sudden, she became affected with hypochondriacal melancholia, and six weeks afterwards died of gangrene in both feet.

Hypochondriacal delirium is thus not only a mere premonitory symptom of certain forms of paralysis, but it is a serious symptom, and one very unfavorable in prognosis.

In reference to this affection, viz., that of hypochondriacal insanity—viewed as one of the precursors of general paralysis, this being the fact of most practical value in connection with it, the delusion of which we have spoken seem to claim especial attention, as they are sometimes to be detected in patients as yet evincing no indication of paralysis—this supervening at a later period. Such a termination is certainly not invariable; but is so common after this symptom, and the prognosis in such cases is so unfavorable, that considerable importance seems to attach to the subject. Thus Dr. Combes published some remarks on a case of "Lypemanie," with stupor, and other serious symptoms—nothing, however, indicating that at a later period this patient should be attacked with paralysis; and, after fifteen months' residence in this asylum, where he was treated, he was dismissed as cured. In reading Dr. Combes's remarks, I was struck, observes M. Baillarger, with certain of the delusions affecting this patient. He had believed that he was about to die, if indeed not already dead; that his limbs were atrophied, that he had none, etc. These appearing to be good grounds for suspicion, I wrote Dr. Combes to know what had become of the patient. The answer confirmed my suspicions to be correct;—the result having been that, after a year's return to his occupations, he had been attacked with general paralysis. We may see by this example, that, had hypochondriacal delirium been held as a certain precursor of general paralysis, this affection might have been foretold two years before it actually took place.

It may appear strange that one form of insanity should thus be urged as premonitory of paralysis. Singular as it may seem, however, it is not the first time that such a doctrine has been urged. Since the writings of Bayle, no medical man doubts the fact of certain forms of insanity, such as the ambitious form, being symptomatic of approaching paralysis. And if one form of delirium be held, in mania or monomania, as indicating the advent of paralysis, there seems no reason why this particular hypochondriacal form should not serve the same purpose, and with equal certainty, in melancholia.

We do not attempt explaining these facts; and, we may add, it seems useless to do so, either here or in the case of ambitious insanity. One point connected with the ambitious form may be mentioned; and that is, the relative frequency of general paralysis among females in different ranks of society. While this malady is equally common among males of all classes, among women it is not so. It is very common among the poor, and very rare among the rich. It would appear, however, that this circumstance has been forgotten by those who would explain the greater number of cases of ambitious insanity as induced by ideas of speculation—by the desire of suddenly arriving at honors and fortune.

In conclusion, it appears evident that hypochondriacal no less than ambitious insanity may, in different circumstances, be considered as a prognostic of general paralysis. The intention of the present paper has accordingly been to direct attention more particularly to the latter of these forms. As for the first, I have frequently had occasion, before now, to refer to it in all its remarkable psychological characters.—*Gazette des Hopitaux*, Sept. 1860, and *Edinburgh Medical Journal*, Dec. 1860.

Perversion of the Mental and Bodily Faculties as premonitory symptoms of General Paralysis. By M. Brierre de Boismont.—This paper, read at the meeting of the "*Académie des Sciences*," Sept. 24, 1860, may be considered as a sequel to that of M. Baillarger, read at its previous meeting, and just noticed. After alluding to the medico-legal aspects of such cases as are characterized by a tendency to theft and other criminal propensities, the author states that his observations have been carefully collected from a hundred examples falling under his own care, and respecting which he had already communicated to the *Société Médico-Psychologique* those alterations in character and disposition throwing light upon the question under discussion.

According to his observation, the most frequent symptoms —in fact, what occur in three-fourths of such cases—are, great irritability, movements of impatience, anger, and violence. In a much smaller proportion of cases, the disease assumes on the other hand, characters of indolence, and apathy, and gentleness. Such patients are reasonable and well-conducted; but between their words and actions there is an insurmountable discrepancy observable.

In place of either of these states of mind, or along with either of them, we frequently find perversion of the moral faculties: individuals who until then had been of unimpeachable character, suddenly becoming irreligious, immoral or dishonest. These indications are important to be observed, as they are often unsuspected by any one as being connected with the diseased condition existing. The propensity to steal is, perhaps, of all others the commonest among this class of symptoms, and seems to some extent connected with that peculiar flow of spirits common in such patients, and evinced in their delusion that they are rich, powerful, and lords of all that they see—a state of mind which, in its results, sometimes entails the most painful consequences.

It is thus certain that the greatest change in the character and conduct is often observed in connection with general paralysis, giving rise to acts of an eccentric or reprehensible nature. Such acts are, no doubt, frequently to be met with as ordinary manifestations of the disposition, but their sudden and unaccountable accession results from mental disease, and is especially connected with general paralysis: they are its premonitory smptoms—*avant-courriers*, as they have been styled by Dr. Forbes Winslow, in his work "On Obscure Diseases of the Mind and Brain."

Our principal reliance in the diagnosis of such cases must rest on the general bearings of the disease. In the greatest number of instances where sudden alteration in character, disposition, and conduct become apparent, there is reason to fear the accession of general paralysis; if the age is from 35 to 45 years, and excess of some kind, such as sexual or intellectual excess, and hereditary predisposition can be added, the prognosis becomes all the more certain.

Besides these characteristic symptoms, we must not lose sight of a very common occurrence connected with them,—that is the frequency of attacks of the nature of cerebral congestion. This may occur in the shape of a transient stunning sensation or giddiness, and pass off without attracting much notice; but it does so more commonly, and is of serious importance. Such congestions entail weakening of the intellectual faculties, loss of memory, and absence of mind. The mind loses its ordinary power. If the patient resume his occupation, and take to any work requiring application, the difference is at once observable in his capability of conducting it. His benevolence is greater than usual; there is a confidence betraying itself in his speech, which foretells the advent of insanity in its ambitious form. On the other hand, but less frequently, there is a state of dejection, the tendency to melancholia and hypochondriasis.

But the disorders of the muscular system are the key or touchstone for our guidance in this disease. One of these is of much importance, and manifests itself in a passing, transient trembling of the lips; a scarcely perceptible embarrassment in speech; a hesitation in pronouncing a certain word or letter, which does not occur except at long intervals. Taken by itself, this symptom may not be determinative, although it is of great assistance: but if it be added to diminution of motive power, such as may be observed in asking the patient to grasp one's hand, or his own limb, the certitude becomes increased tenfold. To these symptoms may be added, inequality of the pupils, exaltation or failure of the sexual functions, diminution of the cutaneous sensibility, tremors of the muscles, and the results of analysis of the urine, and the indications afforded by means of electricity. We have also, in many cases, adds the author, observed paralysis of the sixth pair, amaurosis and attack of deafness, precede by several years the occurrence of general paralysis, and serve as the means for its prognosis.—*Gazette des Hopitaux*, Oct. 1860, and *Edinburgh Medical Journal*, Dec. 1860.

